# Family planning of infertile couples: a systematic review of intentions regarding parenthood and return to ART

**DOI:** 10.1093/humrep/deaf239

**Published:** 2025-12-22

**Authors:** Letizia Li Piani, Giovanna Esposito, Marco Reschini, Jacques Donnez, Fabio Parazzini, Edgardo Somigliana

**Affiliations:** Department of Clinical Sciences and Community Health, Dipartimento di Eccellenza 2023-2027, University of Milan, Milan, Italy; Infertility Unit, Fondazione IRCCS Ca’ Granda Ospedale Maggiore Policlinico, Milan, Italy; Department of Clinical Sciences and Community Health, Dipartimento di Eccellenza 2023-2027, University of Milan, Milan, Italy; Fondazione IRCCS Ca’ Granda Ospedale Maggiore Policlinico, Milan, Italy; Infertility Unit, Fondazione IRCCS Ca’ Granda Ospedale Maggiore Policlinico, Milan, Italy; Société de Recherche Pour L’infertilité, Université Catholique de Louvain, Brussels, Belgium; Department of Clinical Sciences and Community Health, Dipartimento di Eccellenza 2023-2027, University of Milan, Milan, Italy; Department of Clinical Sciences and Community Health, Dipartimento di Eccellenza 2023-2027, University of Milan, Milan, Italy; Infertility Unit, Fondazione IRCCS Ca’ Granda Ospedale Maggiore Policlinico, Milan, Italy

**Keywords:** infertility, ARTs, fertilization *in vitro*, reproductive behaviour, birth order

## Abstract

**STUDY QUESTION:**

What motivations and barriers influence family planning decisions among infertile individuals?

**SUMMARY ANSWER:**

In studying the family planning of infertile couples, this review found that a significant gap persists between desired and achieved family size.

**WHAT IS KNOWN ALREADY:**

While ART has traditionally focused on live birth rate (LBR) as a primary success parameter, growing attention has been paid to whether treatments help couples achieve their desired family size. Evidence suggests that many infertile couples do not return to ART for subsequent children, despite having cryopreserved embryos available.

**STUDY DESIGN, SIZE, DURATION:**

This review was conducted as a systematic review following PRISMA guidelines. A comprehensive search strategy was developed and implemented across PubMed and Embase databases, covering studies published in English up to May 2025. The search combined free text terms and MeSH/Emtree terms related to ‘infertility’ and ‘family planning’.

**PARTICIPANTS/MATERIALS, SETTING, METHODS:**

We included observational studies reporting outcomes related to family size, return to ART, or intentions for subsequent children. Two reviewers independently performed screening, data extraction, and quality assessment using the Newcastle–Ottawa Scale.

**MAIN RESULTS AND THE ROLE OF CHANCE:**

Of 2495 screened records, 9 studies were included. Across contexts, infertile couples consistently reported smaller family sizes compared to fertile ones. Return rates to ART for a second child ranged from 25 to 50%, even among those with cryopreserved embryos. Factors associated with return included younger age, availability of embryos, and previous treatment characteristics. However, emotional, financial, and social burdens often discouraged further ART use. Success rates for second ART pregnancies varied, with cumulative LBRs between 38 and 88%, depending on treatment strategy and prior history.

**LIMITATIONS, REASONS FOR CAUTION:**

Scarcity of evidence and high heterogeneity across studies, including differences in design, populations, outcomes, and type of ART, may have limited comparability of the studies.

**WIDER IMPLICATIONS OF THE FINDINGS:**

The low return rate to ART highlights unmet needs in post-treatment support and counselling. Future research should explore the psychosocial, economic, and systemic barriers that prevent couples from pursuing their reproductive goals, enabling more patient-centred care in reproductive medicine.

**STUDY FUNDING/COMPETING INTEREST(S):**

Open access funding was provided by Università degli Studi di Milano within the CRUI-CARE Agreement. This study was in part supported by the Italian Ministry of Health-Current Research IRCCS. E.S. reports receiving grants from Ferring and honoraria for lectures from Merck-Serono, IBSA, and Gedeon-Richter. J.D. has received consulting fees from ObsEva, Gedeon Richter, and Theramex and was a member of the scientific advisory board of ObsEva and Preglem until 2023. L.L.P. reports participation in a training course sponsored by Gedeon Richter, during which she received medical writing assistance for this paper as part of the training course. The remaining authors declare no competing interests.

**REGISTRATION NUMBER:**

n/a.

## Introduction

In recent years, while the primary focus of assisted reproductive technology (ART) has been on ensuring the safety, efficacy, and accessibility of treatments (von Estorff *et al.*, 2024), attention has increasingly shifted towards evaluating broader success factors, beyond just live birth rates (LBRs) (García *et al.*, 2020; Chen *et al.*, 2025). A critical aspect now under consideration is the alignment between ART outcomes and the reproductive goals of couples. In fact, although LBR serves as a key measure of ART success, it does not fully capture whether couples are achieving their desired family size (Habbema *et al.*, 2015).

Emerging evidence suggests that many infertile couples who undergo ART tend to have only one child and not to return to fertility clinics for additional pregnancies, despite having embryos or blastocysts cryopreserved and available (Paul *et al.*, 2020). This finding raises important questions about the factors influencing couples’ decisions not to pursue further children (Somigliana *et al.*, 2023). Determinants such as financial constraints, emotional strain, and social pressures may all play a role, but this area still represents a gap in knowledge (Somigliana *et al.*, 2023). For instance, there is scant evidence on the proportion of women conceiving a second child naturally.

Evidence on the utilization of available frozen embryos after a live birth and the motivations behind fertility decisions remains surprisingly limited. Although initial fertility intentions do not always translate into the actual family size also in the general fertile population (Liefbroer, 2009), understanding the factors behind these choices among infertile subjects is key to developing supportive interventions that can better align with couples’ evolving needs and aspirations (Schoen *et al.*, 2000; Duthie *et al.*, 2017).

Indeed, the limited number of studies in this field, despite the widespread use of ART, highlights a significant research gap. This systematic review aims to synthesize the current evidence on family planning in infertility, exploring actual and/or desired number of children of infertile couples, their attitudes to returning to ART, and the success of these further attempts.

## Materials and methods

### Data sources and search strategy

This review was conducted using a systematic approach to identify and evaluate relevant literature, in accordance with the Preferred Reporting Items for Systematic Reviews and Meta-Analyses (PRISMA) guidelines (Page *et al.*, 2021). A comprehensive search strategy was developed and carried out across PubMed and Embase. The search string included both free text terms and Medical Subject Headings (MeSH) in PubMed, as well as Emtree terms in Embase, focusing on the topics of ‘infertility’ and ‘family planning’ (Supplementary Table S1). Reference lists of relevant articles were also examined for additional studies not captured by the initial search.

### Review process and selection

Selection criteria were defined prior to the search and included studies published in English, up to 29 May 2025, addressing the fertility attitudes and subsequent decisions among infertile couples. Specifically, observational studies (including retrospective, prospective, and cross-sectional studies) were selected if they reported outcomes related to family planning, such as the number of children, intention of having a second (or higher order) birth, return rate to ART, and conception of a second (or higher order) child after ART. One study investigating only self-reported intention to have a second child (Lau *et al.*, 2018) was also considered, as it provided valuable insights into important aspects of family planning and reproductive decision-making after ART that are directly relevant to the scope of this review. Reviews, case reports, case series, letters, editorials, and animal studies were excluded. Articles written in languages different from English were also excluded.

First, two reviewers (L.L.P. and G.E.) screened all articles to identify potentially eligible studies, and then after reading the abstract and the full text, the authors were able to define which articles were relevant. Information was extracted from the selected papers by the same authors (L.L.P. and G.E.), focusing on key aspects such as study design, sample size, outcomes and major findings. A third author (E.S.) was involved to solve potential discrepancies in study inclusion.

### Quality assessment

Two authors (L.L.P. and G.E.) independently assessed the methodological quality of the included studies using the Newcastle–Ottawa assessment tool, focusing on the three criteria: population selection, comparability, and outcome assessment (Wells *et al.*, 2000). Disagreements were resolved through consultation with a third author (E.S.). An adapted version of the assessment tool was used for cross-sectional studies by omitting the follow-up evaluation.

## Results

### Study characteristics

**Figure 1.**
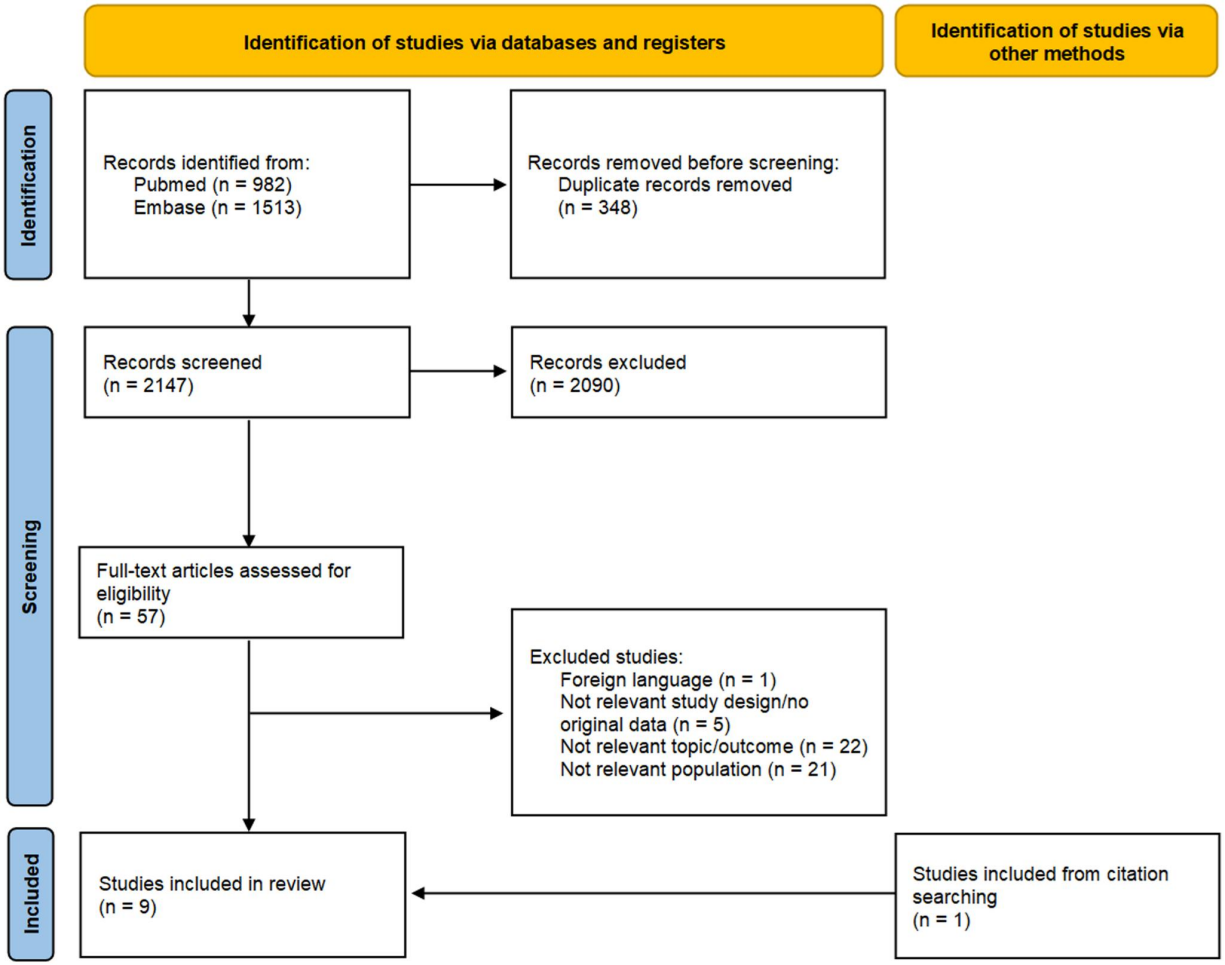
Flowchart for the study selection process.

A total of 982 and 1513 records were identified in PubMed and Embase, respectively. After removing duplicates (n = 348), 2147 titles and abstracts were screened. Of the 57 full-text articles screened for eligibility, 9 were included (Fig. 1) (Guerif *et al.*, 2003; Breyer *et al.*, 2010; Cook *et al.*, 2013; Luke *et al.*, 2013; Lau *et al.*, 2018; Paul *et al.*, 2020; Choi *et al.*, 2023; Esposito *et al.*, 2024; Limena *et al.*, 2024).

Table 1 provides a detailed summary of the characteristics of the included studies. There were five retrospective studies (Cook *et al.*, 2013; Luke *et al.*, 2013; Choi *et al.*, 2023; Esposito *et al.*, 2024; Limena *et al.*, 2024), two prospective studies (Guerif *et al.*, 2003; Paul *et al.*, 2020), and two cross-sectional studies (Breyer *et al.*, 2010; Lau *et al.*, 2018). Three studies were conducted in the USA (Breyer *et al.*, 2010; Cook *et al.*, 2013; Luke *et al.*, 2013), two in Italy (Esposito *et al.*, 2024; Limena *et al.*, 2024), two in Australia (Paul *et al.*, 2020; Choi *et al.*, 2023), one in France (Guerif *et al.*, 2003), and one in China (Lau *et al.*, 2018).

**Table 1.** Summary of the characteristics of the studies investigating family planning in infertility.

Author, year	Country, period	Study design	Population	Comparison	Outcome	Main findings
***Return rate to ART and/or success rate in achieving subsequent pregnancies***						
Guerif *et al.*, 2003	France, 1991–1997	Prospective cohort study	222 couples who achieved a successful pregnancy after DI with frozen sperm	Not applicable	CLBR with DI	CLBR: 65.3% (with a LB rate per cycle of 14.8%) Mean age of women: 32.7 ± 3.0 years
Cook *et al.*, 2013	USA, 2000–2010	Retrospective study	1070 fresh donor oocytes IVF cycle	Not applicable	Return for FET and success rate	FET in the subset of women had a previous successful IVF cycle and had frozen embryos: 25% with 37.8% of women who conceived
						No difference of age between women who returned and those who did not (42.9 ± 5.1 vs 43.1 ± 4.7, *P* = 0.54)
Luke *et al.*, 2013	USA, 2004–2010	Historical cohort study	297 635 women undergoing ART	Returned to ART *versus* not returned to ART	Return rate and LB rate for cycle	LB rate for cycle (1–5), respectively: 34.0, 32.6, 30.6, 29.4, and 27.3% Mean return rate: 25.2%, with a decreasing trend according to age (27.6% in women <30 years; 24.7% in 31–37, 22.2% in 38–40, 18.4% in over 40 years)
Paul *et al.*, 2020	Australia, 2009–2015	Prospective population-based cohort study	35 290 women who had a previous autologous ART-mediated LB	Returned to ART *versus* not returned to ART	Return rate to ART and CLBR	Return rate: 43.4% CLBR: 60.9–88.1% in women who recommenced treatment with a frozen embryo and 50.5–69.8% in women who underwent a new stimulation cycle Median age of women who returned to ART treatment was 36 years [32–38] After adjusting for covariates, women who returned to ART treatment were more likely to be younger (adjusted OR = 0.78 for 35–39 years and adjusted OR = 0.56 for 40–44 years compared to women under 30 years)
Choi *et al.*, 2023*	Australia, 2003–2017	Longitudinal population-based birth cohort study	481 866 first-time mothers	IVF *versus* OI–IUI *versus* subfertile mothers *versus* natural conception	CLBR after natural conception, and CLBR after ART-conception	CLBR (within 5 years) after natural conception in women ART: 27% CLBR (within 5 years) after natural conception in women with other conceptions (52.9–68.8%) CLBR (within 5 years) after ART-conception in in women ART: 42.4% CLBR (within 5 years) after ART-conception in in women OI-IUI: 24.1% IVF mothers: 34.6 ± 4.6; OI-IUI mothers: 31.0 ± 4.4 years; natural mothers: 28.7 ± 5.4 years
Esposito *et al.*, 2024	Italy, 2007–2021	Historical cohort study	431 333 women with natural first births and 16 837 women with ART-mediated first births	Women who had a first ART-mediated birth *versus* women who had a first natural birth	Probability and mode of conception of a second birth	Among women who had a first naturally conceived birth, the probability of having a second birth were 1.1% and 59.3% after ART and natural conception, respectively. The corresponding values were 11.5% (HR = 15.12, 95% CI: 13.99–16.35) and 25.2% (HR = 0.52, 95% CI: 0.50–0.54) for women who underwent ART to have their first live birth.
Limena *et al.*, 2024	Italy, 2013–2021	Retrospective cohort study	374 nulliparous women under 40 years	Returned to ART *versus* not returned to ART	Return rate to ART and CLBR	Return rate: 50.3% CLBR (within 5 years): 56.4% Women who returned were younger than those who did not (34 [IQR: 32–36] years versus 36 [IQR: 34–38] years)
***Family size***						
Breyer *et al.*, 2010	USA, 2002–2003	Cross-sectional survey study	4928 men and 7643 women aged 15–45 years	Infertile *versus* fertile (status self-reported)	Family size	Number of children in infertile females: 2.0 ± 1.0; in fertile females: 2.2 ± 1.2 In women who sought reproductive assistance OR of having more than one child: 0.66 (0.48–0.90), less when male infertility was declared Infertile men and women tended to be older than fertile counterparts at first child (women: 25.3 ± 5.0 vs 21.9 ± 4.9; men: 28.7 ± 5.1 vs 25.2 ± 5.2 years) Number of children in infertile males (9.7%): 1.8 ± 0.7; in fertile males: 2.1 ± 1.2 In males who declared difficult or not possible to father a child OR of having more than one child: 0.34 (0.13–0.86)
Choi *et al.*, 2023*	Australia, 2003–2017	Longitudinal population-based birth cohort study	481 866 first-time mothers	IVF *versus* OI–IUI *versus* subfertile mothers *versus* natural conception	Number of children	ART mothers tended to have smaller families: 2.54 children in case of IVF; 2.98 in OI–IUI, 2.78 in subfertile women and 3.23 in natural conception. The fertility recovery phase for fertility gap was reached in OI–IUI mothers (30.3 years) compared with IVF (36.4 years) and subfertile ones (37.8 years).
Lau *et al.*, 2018	China, 2016	Cross-sectional survey study	974 married women aged 20–45 years	Infertile *versus* fertile (status self-reported)	Intention to have a second child	Women with intention to have a second child (infertile *versus* fertile): 76.6% *versus* 61.9%, *P* < 0.01. Women mean age (whole cohort): 32.0 ± 4.9 years; Men mean age (whole cohort): 34.1 ± 5.5 years Advanced age and childcare costs were considered as the heaviest potential barrier to having a second child for those who expressed their intention not to have it (106/299). Reduction of the intention of having a second child was reported for female older age (adjusted OR = 0.95) and male one (adjusted OR = 0.97), longer marriage (adjusted OR = 0.97) and a full-time occupation (adjusted OR = 0.64). In case of women with a child, second-child intention was hampered by full-time occupation (OR = 0.48), and supported by higher ideal parity (OR = 11.38)

CLBR, cumulative live birth rate; DI, donor insemination; FET: frozen embryo transfer; IQR, interquartile range; HR, hazard ratio; LB, live birth; OI, ovulation induction; OR, odds ratio.

* The study Choi *et al.*, 2023 contributes to both identified sub-themes.

In general, studies included women who underwent (or a number of cycles of) different types of ARTs (Guerif *et al.*, 2003; Cook *et al.*, 2013; Luke *et al.*, 2013; Paul *et al.*, 2020; Esposito *et al.*, 2024; Limena *et al.*, 2024); in one case, they were also compared with fertile women who conceived naturally (Choi *et al.*, 2023). Two other studies compared fertile and infertile populations, with infertility status self-reported (Breyer *et al.*, 2010; Lau *et al.*, 2018).

### Quality assessment

Results obtained from the quality assessment are summarized in Supplementary Table S2. Only four out of nine studies compared infertile and fertile populations (Breyer *et al.*, 2010; Lau *et al.*, 2018; Choi *et al.*, 2023; Esposito *et al.*, 2024). Two studies (Guerif *et al.*, 2003; Cook *et al.*, 2013) included selected populations of couples undergoing ART with donor gametes. Two other studies considered self-reported fertility status (Breyer *et al.*, 2010; Lau *et al.*, 2018).

### Family size: actual and desired number of children

According to the literature, there is a consistent pattern across different contexts: infertile couples generally have fewer children compared to their fertile counterparts.

A US cross-sectional survey conducted in the early 2000s reported that for both men (1.8 compared to 2.1) and women (2.0 compared to 2.2), infertile people had fewer children on average than fertile ones (Breyer *et al.*, 2010). In a longitudinal population-based birth cohort study conducted in Australia, mothers who conceived through IVF had an average of 2.54 children, compared to 2.98 for those undergoing ovulation induction (OI) or intrauterine insemination (IUI), 2.78 for subfertile women, and 3.23 for those who conceived naturally (Choi *et al.*, 2023).

Similarly, a study based on four European registries found that couples whose first pregnancy took at least 12 months to conceive had a significantly higher risk of not having a second child (OR = 1.8 [95% CI: 1.58–2.04]) or a third child (OR = 1.6 [95% CI: 1.35–1.87]), regardless of the mother’s age (Joffe *et al.*, 2009). However, we did not include this study in our systematic review because it focused on biological fertility, using time to pregnancy as a proxy for subfertile status, and did not consider the mode of conception.

Regarding the intention to have a second child, a study investigating childbearing attitudes in China found that infertile women were more likely to want a second child than fertile women (77% versus 62%), with infertile women who already had a live child reporting the highest intentions (Lau *et al.*, 2018).

### Return rate to ART after live birth

Several studies reveal a clear trend: only a modest proportion of infertile couples return to ART for subsequent children, a pattern observed across studies and contexts.

A retrospective monocentric Italian study reported that only 50% of patients (188/387) returned for further ART treatments after a median of 2.2 years, despite some having cryopreserved embryos. Of those who did not return, 30% expressed interest in having a second child but did not undergo ART and tried unsuccessfully to conceive naturally (Limena *et al.*, 2024). Even in Australia, known for its family-friendly policies, only 43% of women (15 325/35 290) who had a live birth from their first IVF cycle returned for a second attempt within 2 years (Paul *et al.*, 2020). A lower return rate to ART of 25% was also reported in a US study including around 300 000 women between 2004 and 2010 (Luke *et al.*, 2013).

These results are consistent with findings that couples are more likely to rely on ART for their first child than for subsequent children. In a US study involving 1070 fresh donor oocyte IVF cycles, only 25% of those who conceived during their initial cycle (99/403) returned to use their frozen embryos, compared with a 65% return rate among those with unsuccessful results (140/215) (Cook *et al.*, 2013). Likewise, an Italian population-based analysis of over 1 million deliveries showed that couples were significantly less likely to pursue additional ART cycles after successful births, regardless of the modality of conception. The probability of undergoing ART to achieve a second birth compared to the first one was 0.14 (95% CI: 0.13–0.15), after adjusting for age, education, and nationality (Esposito *et al.*, 2023); however, this study was excluded from our systematic review because it did not directly address the outcome of interest. It did not report the number of children or the return rate to ART among infertile women but only provided data on the proportion of ART use for first- and second-order children.

### LBR after ART in achieving subsequent pregnancies

In addition to the rate of return to ART, some studies examined success rate in obtaining a second child from ART, measured as the cumulative live birth rate (CLBR) and/or the LBR per cycle.

The CLBR within 5 years in the Italian monocentric study was 56% (Limena *et al.*, 2024). The Australian study showed a CLBR of 61–88% in women who restarted treatment with a frozen embryo and 51–70% in women who underwent a new stimulation cycle (Paul *et al.*, 2020). In line with this, a French study including 222 couples, who achieved a successful pregnancy after donor insemination with frozen sperm and returned for a new attempt, reported a CLBR of 65% with an LB rate per cycle of 15% (Guerif *et al.*, 2003). In this study, 10% of patients who did not achieve pregnancy switched to IVF, so the success rate in obtaining a second pregnancy after ART may be underestimated. The CLBR for frozen embryo transfer after a first successful fresh donor oocyte IVF was 38% (Cook *et al.*, 2013). In the US study with the lower return rate, the LB rate per cycle declined from 34% in the first cycle to 27% by the fifth cycle (Luke *et al.*, 2013).

The CLBR varied significantly depending on the method of conception and prior reproductive history (Choi *et al.*, 2023; Esposito *et al.*, 2024). In a longitudinal Australian study, women who had their first birth after ART had a CLBR after IVF of 42% within 5 years, compared with 24% for women who had their first birth after OI or IUI. The CLBR, including natural conceptions, was 27% for those who previously underwent ART and between 53% and 69% for those with other types of conceptions (Choi *et al.*, 2023). In a second Italian population-based analysis, 12% of women who had a first birth with ART had a second birth after ART, compared with only 1% of women whose first birth was naturally conceived (Esposito *et al.*, 2024).

### The role of age in shaping the family size and return rate

Age emerged as a central factor in almost all studies examining both completed family size and the likelihood of returning for further treatment after a live birth.

Regarding family size, male age was more frequently considered in relation to the total number of children, whereas female age was predominantly linked to the decision to return for subsequent treatment. Breyer *et al.* observed that infertile men and women tended to be older than their fertile counterparts at first child (women: 25.3 ± 5.0 vs 21.9 ± 4.9; men: 28.7 ± 5.1 vs 25.2 ± 5.2 years) (Breyer *et al.*, 2010). Similarly, the impact of delayed childbearing was described across different modes of conception: the recovery phase for the fertility gap was reached at 30.3 years in the case of OI–IUI mothers, earlier than for IVF or subfertile mothers (36.4 and 37.8 years, respectively). Indeed, none of these groups closed the fertility gap by the end of their reproductive lives, leading to a permanent difference in completed family size, unlike women with natural conception (Choi *et al.*, 2023).

In terms of return rates after ART success, several studies highlighted the negative impact of advancing age. Luke *et al.* (2013) reported a decreasing trend in return rates with age (27.6% in women <30 years, 24.7% at 31–37, 22.2% at 38–40, and 18.4% >40 years). In an Australian cohort, women who returned to treatment were more likely to be younger, with adjusted odds ratios (ORs) of 0.78 for women aged 35–39 and 0.56 for those aged 40–44 compared with women under 30 (Paul *et al.*, 2020), while a difference of 2 years was observed in the Italian setting in median age (34 [IQR: 32–36] vs 36 [IQR: 34–38] years) (Limena *et al.*, 2024). Similarly, age differences were observed by type of treatment: women undergoing IVF were older than those treated with OI–IUI (34.6 ± 4.6 vs 31.0 ± 4.4 years) and those conceiving naturally (28.7 ± 5.4 years) (Choi *et al.*, 2023). Furthermore, advanced maternal or paternal age was cited as the strongest barrier to a second child by 35% of couples who expressed their intention not to attempt another pregnancy (adjusted OR = 0.96) (Lau *et al.*, 2018). The only exception to this pattern was reported by Cook *et al.* (2013), who found no significant age difference between returners and non-returners (42.9 ± 5.1 vs 43.1 ± 4.7 years, *P* = 0.54).

## Discussion

### Why infertile couples tend to have fewer children then their fertile counterparts?

According to the literature, infertile couples generally have fewer children than fertile ones (Joffe *et al*., 2009; Breyer *et al.*, 2010; Choi *et al.*, 2023). The diagnosis of infertility and a longer time to pregnancy are associated with a reduced likelihood of having a larger family, demonstrating a biological role in determining family size. These elements are both causes and consequences of advanced maternal age, often due to postponed childbearing, which further contributes to a reduced family size. In fact, women undergoing ART for the first child are generally older compared with their fertile counterparts (Breyer *et al.*, 2010; Choi *et al.*, 2023), narrowing an already limited reproductive window for additional children. Therefore, age acts as a crucial biological limitation and a sociocultural marker of delayed childbearing, leading to the fertility gap in infertile couples that persists until the end of their reproductive years (Choi *et al.*, 2023).

However, the decision to have or plan a second child is influenced by a wide range of factors beyond age, even among couples without fertility problems. Childbearing decision-making is a complex process involving individual, relational, and contextual factors that span from marital status to interpersonal network, urban residence, job stability, and broader cultural and societal frameworks (ESHRE Capri Workshop Group, 2001; Joffe *et al.*, 2006; Leridon and Slama, 2008; Sundaram *et al.*, 2020; Hashemzadeh *et al.*, 2021; Cusatis *et al.*, 2023). Interestingly, among young adults who have not started their family journey yet, surveys consistently report a desired family size of around two or more children, at an age with a known decrease of female fertility (Vassard *et al.*, 2016; Place *et al.*, 2025). However, the number of children that these intentions result in is often higher than the number of children that are actually achieved, reflecting a general fertility gap that is even more pronounced among infertile couples (Nitsche and Hayford, 2020). For infertile couples, the determinants of this decision may be more complex, influenced not only by these common factors but also by the emotional, physical, and financial burden of infertility treatments, the psychological impact of infertility itself and previous unsuccessful attempts, as well as concerns about the health and well-being of both the parents and potential offspring (Soullier *et al.*, 2011; Gameiro *et al.*, 2012; Kiesswetter *et al.*, 2023; Limena *et al.*, 2024).

Moreover, it must be emphasized that intentions for conception may play a crucial role (Lakha and Glasier, 2006). In fertile couples, conception can occur despite low rates of intentions while, in infertile couples, couples need to actively pursue pregnancy, and a low intention rate may negatively influence referral to ART centres. Furthermore, attention should be paid to the potential role of regrets about either action or inaction, which could weigh particularly on infertile couples and also differ within the couple (Greil *et al.*, 2022; Cusatis *et al.*, 2023). These feelings of ambivalence and regret are compounded by multiple and potentially conflicting priorities during their family journey (Duthie *et al.*, 2017) as well as by the specific psychological needs that might be likely underestimated by the IVF clinical staff (Scognamiglio *et al.*, 2025). Altogether, these elements form a framework of complex experiences and constraints faced by infertile couples that should be explored with a more comprehensive and nuanced approach.

For example, in the Australian study (Choi *et al.*, 2023), socioeconomic status emerged as a strong determinant of family size. In lower socioeconomic areas, mothers conceiving through ART were significantly less likely to achieve the same family size as naturally conceiving mothers, with a fertility gap of 0.83 fewer children. Although the difference in family size between ART and naturally conceived mothers persisted at all socioeconomic levels, the impact of socioeconomic status was particularly pronounced: ART mothers from higher socioeconomic areas had a smaller fertility gap of 0.43 fewer children. This disparity may be due to the high financial cost of ART treatments, which may be less affordable for families from lower socioeconomic backgrounds. In addition, limited access to healthcare resources, reduced fertility awareness, and greater stressors associated with socioeconomic disadvantage may further hinder the ability of low-income couples to initiate or complete their ART journey (Choi *et al.*, 2023). This finding aligns with a broader framework in which socioeconomic status, including conditions experienced during childhood, has been recognized as relevant in shaping fertility intentions (Hua *et al.*, 2025). This suggests that policymakers must boost economic support for low-income families to mitigate low fertility rates caused by childhood hardships (Hua *et al.*, 2025).

The US cross-sectional survey (Breyer *et al.*, 2010) reports that, in addition to socioeconomic factors, the cause of infertility plays a role in determining the number of children. The authors found that male infertility had a greater impact on family size than female infertility: women whose partner had an abnormal semen analysis had the smallest number of offspring (mean 1.5) among the various infertility definitions analysed. It is possible that the biological causes of male infertility are more difficult to address or that treatments are less frequently sought compared to those with female infertility (Breyer *et al.*, 2010). In support of this, men in the USA are less likely than women to use reproductive services (Anderson *et al.*, 2009). In addition, a diagnosis of male infertility is known to negatively impact marital relationships, which can add emotional burden, discouraging further reproductive attempts (Smith *et al.*, 2009). Also, different family-building priorities within the couple might have a role in this finding (Duthie *et al.*, 2017).

Some studies in the past have reported that acceptance of multiple pregnancies as a result of ART treatment increases with age and duration of infertility (Child *et al.*, 2004). In line with this, when looking at the desired number of children rather than the actual number, women who have experienced an infertility diagnosis and/or identify as infertile, however, express greater desires for a second child and a higher ideal number of children (Shreffler *et al.*, 2016). A more recent study from China found that infertile couples were less confident about achieving their parity goals and experienced greater emotional distress, despite reporting higher ideal family sizes (Lau *et al.*, 2018). This finding highlights the potential role of every condition of uncertainty, such as infertility diagnosis, in narrowing the vision of family size, similar to other events that are associated with instability, such as economic crises or public health emergencies, like the recent COVID-19 pandemic (Vignoli *et al.*, 2020; Guo *et al.*, 2025). Pronatalist attitudes, unexplained infertility, previous childbirth, and religion were associated with greater intentions among infertile women, even when they were less confident about their success rate. In contrast, among fertile women, older age, full-time work, and lower confidence in achieving parity goals decreased intention to have a second child (Lau *et al.*, 2018). A recent longitudinal population-based study of fertility decision-making in uncertain conditions found that, while their underlying orientation towards having children remains largely unchanged, individuals experiencing infertility tend to lower their perceived likelihood of having children in the future. This suggests that, despite reduced expectations, couples may still pursue their desired future of parenthood (Lazzari, 2025).

Not least, the limited effectiveness of ART should be weighed as a crucial determinant for the definition of the family size. Most studies consistently indicated that cumulative outcomes after a second ART course are lower than those reported for first births, reflecting both the biological limitations of advancing maternal age and the cumulative burden of treatment. CLBRs ranged from 38% after donor oocyte IVF (Cook *et al.*, 2013) to 55% in women resuming treatment with new stimulation and over 80% for those with cryopreserved embryos (Paul *et al.*, 2020), with a decreasing trend with increasing cycle number; from the first to the fifth cycle, the live birth decreased from 34 to 27% (Luke *et al.*, 2013). Importantly, the likelihood of enlarging the family also depends on the mode of conception for the first child, as CLBRs were lower in the case of the use of ART for the first birth compared with those who conceived naturally for the first time (Choi *et al.*, 2023; Esposito *et al.*, 2024). These data highlight the modest role of ART in supporting larger family size and emphasize the need for realistic counselling about reproductive planning and for effective strategies to maximize success within the limited reproductive window.

All together, these findings underscore that while ART can support pregnancy achievement, it has only a modest role in solving fertility declines and overall family size definition. These considerations call for broader policies that support individuals in having children, if desired, and promote awareness of age-related infertility.

### What are the determinants of return to ART?

A significant number of couples do not return to ART for subsequent children, even after a success and despite having frozen embryos available (Cook *et al.*, 2013; Luke *et al.*, 2013; Paul *et al.*, 2020; Esposito *et al.*, 2024; Limena *et al.*, 2024). This finding emphasizes the need for further research into the reasons behind couples’ decisions. Understanding these factors is crucial to developing targeted strategies that better address the specific needs and concerns of couples who miss the opportunity for a second child. The factors influencing the return rate for additional ART cycles among infertile couples are multifaceted, with recent research shedding light on key determinants.

Age has emerged as a crucial factor to define the return rate (Luke *et al.*, 2013; Paul *et al.*, 2020; Esposito *et al.*, 2023; Limena *et al.*, 2024). In an Italian retrospective analysis, younger mothers were more prone to undergo ART for a second birth. ART was found to play a greater role in the conception of the first birth compared to the second, with ART-mediated first births being four times more common than ART-mediated second births (Esposito *et al.*, 2023). Another Italian study found that of couples who did not pursue further ART after a first successful attempt, 36% gave up on parenthood, 34% conceived naturally after the first ART-mediated birth, and 30% still desired a second child, suggesting a potential gap between intention and action (Limena *et al.*, 2024). Women interested in having a second child but not using ART were compared to those seeking a second pregnancy using ART, and similar characteristics were found, except for older age in the non-returning group (Limena *et al.*, 2024). In line with this, a third Italian study, limited to pregnancies carried to term and thus unable to explore intentions behind unsuccessful attempts, found that among women with a first ART-mediated birth, the probability of having a second birth after natural conception was 25% (Esposito *et al.*, 2024). Even regarding the intentions, a wish for a second child was reported less frequently in the case of older female age (adjusted OR = 0.95) and older male age (adjusted OR = 0.97) (Lau *et al.*, 2018). The impact of age is even more significant when considering its operational effect on the likelihood of family completion (Habbema *et al.*, 2015). Nevertheless, most studies ultimately focused on maternal age, which reflects not only its biological role in defining reproductive potential but also a broader framework that tends to overlook the role of the partner. The assumption of homogamy, namely the tendency to choose a partner with similar characteristics, background, and values, may ignore that male age and his intentions play a significant role in shaping family size (Mussino and Uggla, 2025) and return rate for IVF couples. As international studies have shown that 25–50% of all couples disagree in wanting a child, further research should explicitly address paternal age and intentions as an integral part of the family-building dynamics (Doepke and Kindermann, 2019).

In addition to age and the possibility of natural conception following a first ART-mediated birth, other motivations for discontinuing a second ART cycle include adoption or personal reasons, such as relocation, divorce, psychological and emotional stress, and, less frequently, medical reasons (Guerif *et al.*, 2003; Limena *et al.*, 2024). These elements are accompanied by potential effects of external factors in reshaping the commitment of couples to reproductive goals. For example, during the COVID-19 pandemic, the increased availability of time coupled with the reduced workload allowed higher dedication to family building (Forte *et al.*, 2024), contradicting other findings that reported a negative effect of public health emergencies (Guo *et al.*, 2025). Therefore, an accurate depiction should encompass contextual factors as well.

Other determinants of return to ART include the availability of surplus frozen embryos (Limena *et al.*, 2024), younger maternal age, nulliparity at the time of the first ART-conceived child, a higher number of oocytes retrieved in the successful cycle, use of ICSI rather than conventional IVF, fresh embryo transfer, pregnancy achieved through single embryo transfer, and multiple ovarian stimulation cycles to achieve the first LB (Paul *et al.*, 2020). Joffe *et al.* suggested that the struggle to conceive may discourage couples from trying again, and the emotional trauma from miscarriage or stillbirth may prevent further attempts (Joffe *et al*., 2009). In addition, births after prolonged conception attempts often involve expensive fertility treatments, which may discourage further attempts. Also, previous twin pregnancies were identified as a factor associated with not returning to ART (Luke *et al.*, 2013).

Although our study focuses specifically on the return rate for a second child, it is helpful to consider this in the wider context of ART discontinuation. Previous research (Scognamiglio *et al.*, 2025), which involved couples seeking their first fertility consultation at a specialized fertility centre, revealed that around 31% of couples did not return after the initial consultation. Reasons for not returning included eligibility for gamete donation, achieving a natural conception, or transferring treatment to another centre. Non-returning women were older and had lower ovarian reserves. Together, these findings highlight that the decision to continue ART is influenced by a combination of clinical factors, prior success, and personal, psychological, and logistical considerations. Recognizing this broader perspective is important for developing strategies to better support couples in achieving additional children if desired.

Not least, it is important to recognize that reproductive intentions can change over time. After having their first child, couples may reconsider their initial preferences considering the practical, emotional, and financial challenges of parenthood. From this perspective, discontinuation of treatment should not automatically be interpreted as premature or as non-compliance, but rather as a legitimate adjustment to reproductive goals. Furthermore, unlike fertile couples who may experience unplanned subsequent pregnancies, couples undergoing fertility treatment generally have greater control over their family size. Consequently, a smaller family size among infertile couples should not necessarily be viewed as an adverse outcome. Indeed, ‘underachievement’ of family size may be the consequence of evolving goals rather than failures in achieving these goals, resulting in a ‘recalibration’ of the initial plan (Nitsche and Hayford, 2020).

Another aspect to consider when interpreting the factors influencing the decision to return to ART is the limited availability of data on naturally conceived second pregnancies following an ART-conceived first birth. Across the studies included in our review, information on naturally conceived subsequent births was either sparse or reported inconsistently. This is a relevant gap, as unassisted conception can be an important factor in shaping couples’ evolving reproductive expectations, intentions, and need to seek ART again. Natural conception after ART, particularly among younger women or those with less severe infertility diagnoses, will reduce the likelihood of returning to treatment by acting as a competing event.

In summary, while current research identifies some key factors influencing return rates for additional ART cycles, significant knowledge gaps persist. Future studies should investigate the specific barriers preventing couples from pursuing further ART treatments when desired and factors that could modify the initial family plan, in order to develop more effective support strategies and improve ART outcomes.

### Limitations

A major limitation of this systematic review is the relative paucity of evidence in the existing literature and a high heterogeneity among the identified studies. This heterogeneity is evident in several key aspects, including differences in study design, variations in populations studied, and the range of reported outcomes. Also, the inclusion of different ARTs, such as donor gametes, further contributes to this heterogeneity. In particular, the lack of consistent or clearly defined outcome measures complicates the establishment of a common metric for comparing results between studies or for drawing reliable conclusions.

### Wider implications

This review highlights several key areas for future research. First, a clear trend has emerged: infertile couples exhibit a low return rate to IVF clinics, despite the availability of frozen embryos. Therefore, a more holistic approach should account for potential differences between an individual’s intended family size, the couple’s shared vision, and their actual intention for family size, which may fluctuate over time due to life events. Additionally, comparing the attitudes of fertile and infertile couples, as well as investigating how IVF impacts family planning, represents a significant unmet research need.

Second, the key factors influencing the decisions of couples to embark on a reproductive journey remain underexplored. Similarly, a deeper insight into the determinants that influence their choice to pursue additional cycles, along with the potential difference of priorities within the couple, could improve counselling practices, offering more realistic guidance on achieving their shared desired family size.

Furthermore, understanding the factors that influence the return rate could be the basis of adequate strategies to prevent ‘family planning burn-out’ and long-term regrets. Identifying perceived barriers that hamper the full realization of couples’ reproductive intentions could lead to more targeted support to help couples achieve their reproductive goals.

## Conclusion

Our review underscores the consistently low return rate of infertile couples to IVF clinics for a second child. A significant gap remains in understanding the key determinants of return rates and long-term reproductive intentions of these couples.

Exploring how ART affects perceptions and decisions around family planning could lead to personalized counselling strategies. Rather than considering discontinuation as premature or undesirable, it is important to recognize that reproductive intentions may change after the birth of a first child and that some couples may choose to adjust or limit their family size deliberately. Counselling approaches should therefore not only aim to reduce barriers to continued treatment but also respect and support couples in revising their reproductive goals, in light of their experiences. In the future, investigating the psychological and logistical challenges in infertile couples and recognizing both partners’ visions and needs will be pivotal in developing targeted interventions that offer more supportive, flexible, and couple-centred treatment options.

## Supplementary Material

deaf239_Supplementary_Table_S1

deaf239_Supplementary_Table_S2

## Data Availability

The data underlying this article are available in the article and in its online supplementary material.
