# Supplementary material for: Family planning of infertile couples: a systematic review of intentions regarding parenthood and return to ART
Source: Hum Reprod. 2025 Dec 22;41(2):204–13. doi: 10.1093/humrep/deaf239 (PMC12864153; doi:10.1093/humrep/deaf239)
Supplement: deaf239_Supplementary_Table_S1 [file deaf239_supplementary_table_s1.pdf]

OXFORD
human reproduction

Human Reproduction, 2025, 1–1
https://doi.org/10.1093/humrep/deaf239
Supplementary Data

Supplementary Table S1. Search strings used for PubMed and Embase (search date: 29 May 2025).

Database	Search string
PubMed	(‘family size’ [All Fields] OR ‘second birth*’ [All Fields] OR ‘second try’ [All Fields] OR ‘second child*’ [All Fields] OR ‘second parenthood’ OR (birth order[MeSH Terms]) OR ‘parenthood intention*’ [All Fields] OR (parenthood [All Fields] AND intention* [All Fields]) OR (reproductive behaviour [MeSH Terms]) OR ‘return rate’ [All Fields])<br>AND<br>(infertility[All Fields] OR ‘assisted reproductive technolog*’ [All Fields] OR ‘in vitro fertilization’ [All Fields] OR IVF [All Fields] OR ‘intracytoplasmic sperm injection’ [All Fields] OR ICSI [All Fields] OR (clinic, infertility [MeSH Terms]) OR (assisted reproductive technologies [MeSH Terms]) OR (fertilization *in vitro* [MeSH Terms]) OR (reproductive medicine [MeSH Terms]) OR (female infertility [MeSH Terms]) OR (male infertility [MeSH Terms]))
Embase	(‘family size’/exp OR ‘family size’ OR ‘second birth*’ OR ‘second try’ OR ‘second child*’ OR ‘second parenthood’ OR ‘birth order’/exp OR ‘birth order’ OR ‘parenthood intention’ OR ((‘parenthood’/exp OR ‘parenthood’) AND (‘intention’/exp OR intention)) OR ‘reproductive behavior’/exp OR ‘reproductive behavior’ OR ‘return rate’)<br>AND<br>(‘infertility’/exp OR ‘infertility’ OR ‘assisted reproductive technolog*’ OR ‘in vitro fertilization’/exp OR ‘in vitro fertilization’ OR ‘IVF’/exp OR IVF OR ‘intracytoplasmic sperm injection’/exp OR ‘intracytoplasmic sperm injection’ OR ‘ICSI’/exp OR ICSI)<br>AND<br>(‘article’/it OR ‘review’/it)

MeSH, Medical Subject Headings; OR, odds ratio.
