# Supplementary material for: Family planning of infertile couples: a systematic review of intentions regarding parenthood and return to ART
Source: Hum Reprod. 2025 Dec 22;41(2):204–13. doi: 10.1093/humrep/deaf239 (PMC12864153; doi:10.1093/humrep/deaf239)
Supplement: deaf239_Supplementary_Table_S2 [file deaf239_supplementary_table_s2.pdf]

OXFORD
human reproduction

*Human Reproduction*, 2025, 1–1
https://doi.org/10.1093/humrep/deaf239
Supplementary Data

**Supplementary Table S2.** Methodological quality of included studies.

| | Selection | | | | Comparability | Outcome | | |
|---|---|---|---|---|---|---|---|---|
| **Study** | ***Representativeness of the exposed cohort*** | ***Selection of the non-exposed cohort*** | ***Ascertainment of exposure*** | ***Outcome of interest was not present at the start of the study*** | ***Comparability of cohorts on the basis of the design or analysis*** | ***Assessment of outcome*** | ***Was follow-up long enough for outcomes to occur?*** | ***Adequacy of follow up of cohorts*** |
| Guerif *et al.*, 2003 | Selected cohort | | * | * | | * | * | * |
| Breyer *et al.*, 2010 | * | * | Self-reported | NA | * | * | * | NA |
| Cook *et al.*, 2013 | Selected cohort | | * | * | | * | * | * |
| Luke *et al.*, 2013 | * | | * | * | | * | * | * |
| Lau *et al.*, 2018 | * | * | Self-reported | NA | * | * | * | NA |
| Paul *et al.*, 2020 | * | | * | * | | * | * | * |
| Esposito *et al.*, 2024 | * | * | * | * | * | * | * | * |
| Limena *et al.*, 2024 | * | | * | * | | * | * | * |
| Choi *et al.*, 2023 | * | * | * | * | * | * | * | * |
